# Identification and Bioaccessibility of Maillard Reaction Products and Phenolic Compounds in Buckwheat Biscuits Formulated from Flour Fermented by *Rhizopus oligosporus* 2710

**DOI:** 10.3390/molecules28062746

**Published:** 2023-03-18

**Authors:** Małgorzata Wronkowska, Wiesław Wiczkowski, Joanna Topolska, Dorota Szawara-Nowak, Mariusz Konrad Piskuła, Henryk Zieliński

**Affiliations:** Institute of Animal Reproduction and Food Research, Department of Chemistry and Biodynamics of Food, Polish Academy of Science, 10-748 Olsztyn, Poland

**Keywords:** buckwheat flours, fungi, fermentation, phenolic compounds, Maillard reaction products, bioaccessibility

## Abstract

The identification and potential bioaccessibility of phenolic compounds using the highly sensitive micro-HPLC-QTRAP/MS/MS technique and Maillard reaction products (MRPs) in buckwheat biscuits formulated from flours, raw and roasted, fermented by *Rhizopus oligosporus* 2710 was addressed in this study after in vitro digestion. The content of the analyzed MRPs such as furosine, FAST index, and the level of melanoidins defined by the browning index was increased in the biscuits prepared from fermented flours as compared to the control biscuits prepared from non-fermented ones. After in vitro digestion higher content of furosine was observed in control and tested biscuits providing its high potential bioaccessibility. The fermented buckwheat flours used for baking affected the nutritional value of biscuits in comparison to the control biscuits in the context of the twice-increased FAST index. More than three times higher value of the browning index was noted in control and tested biscuits after digestion in vitro indicating the high bioaccessibility of melanoidins. Our results showed the presence of ten phenolic acids and eight flavonoids in the investigated biscuits. Among phenolic acids, vanillic, syringic, and protocatechuic were predominant while in the group of flavonoids, rutin, epicatechin, and vitexin were the main compounds in analyzed biscuits. Generally, the lower potential bioaccessibility of phenolic acids and higher potential bioaccessibility of flavonoids was found for biscuits obtained from buckwheat flours fermented by fungi compared to control biscuits obtained from non-fermented flours. Fermentation of buckwheat flour with the fungus *R. oligosporus* 2710 seems to be a good way to obtain high-quality biscuits; however, further research on their functional properties is needed.

## 1. Introduction

Fermentation is one of the oldest processes used by man to obtain products with extended shelf life or with modified sensory properties. Tempe fermentation is one example of the traditional way of processing soybeans into tempeh products, originating in Indonesia. For the production of tempeh, fungi of the genus *Rhizopus* are used, such as *R. oryzae, R. arhizus, R. stolonifer,* and most often *R. oligosporus* [1]. One of the interesting applications of the fermentation process is the production of food enriched with non-enzymatic antioxidants [2]. Qin et al. [3] showed that oat polyphenols may be a food source that could inhibit the formation of advanced glycation end products in vitro. It has been shown that the fermentation of the tempeh of legume seeds may increase the level of compounds showing the activity of scavenging free radicals [4,5]. As was shown in our previous investigation [6,7], the fermentation with *Rhizopus oligosporus* of raw and roasted buckwheat groats enhanced water-soluble vitamins such as thiamine, pyridoxine, and L-ascorbic acid, as well as α-, δ- and γ-tocopherol contents, protein digestibility was improved and higher amount of amino acids and minerals compared with the non-fermented sample was found. Furthermore, the sensory evaluation proved that buckwheat products obtained by fungi fermentation were quite well received by the evaluators.

Buckwheat groats, raw or roasted, are present in the diet of the inhabitants of Central and Eastern Europe. They are usually served like rice after cooking, while raw buckwheat (groat or flour) is used as a substitute for wheat flour in products for people suffering from celiac disease or gluten sensitivity. The literature data indicate that roasting affects the chemical composition and functional properties of buckwheat groats. Zielińska et al. [8] showed the reduction of parent antioxidants as well as the formation of Maillard reaction products after buckwheat roasting. The behavior of some buckwheat ingredients during the digestive process and their bioaccessibility (D-chiro-inositol or quercetin) have been determined and shown by Zieliński et al. [9,10].

Carbonell-Capella et al. [11] presented a definition of bioaccessibility as a fraction of compounds released from the food matrix during the digestion process which can be used for intestinal absorption. The literature data show information about using in vitro models to study the complex multistage process of human digestion [12]. Estimation of the health-benefits potential of functional foods should be connected with bioaccessibility. However, as presented by Carbonell-Capella et al. [11], many factors affect bioaccessibility, such as the composition of the food matrix and its texture, the pH, temperature, and enzymes involved in digestion.

In the present study, the aim was to determine the content and potential bioaccessibility of phenolic compounds and Maillard reaction products in vitro-digested buckwheat biscuits formulated from fermented flours by *Rhizopus oligosporus* 2710.

## 2. Results and Discussion

### 2.1. The Maillard Reaction Indexes and Their Bioaccessibility from Investigated Samples

The profile of the Maillard reaction products in the biscuits obtained from unfermented and fermented by fungi buckwheat flours (raw and roasted) included the level of furosine formed in the early phase of the Maillard reaction [13,14], the fluorescence of tryptophan and the FAST index [14,15], and the level of melanoidins defined by the browning index [14]. All the obtained results for the Maillard reaction products and their potential bioaccessibility are presented in Table 1. Generally, in the biscuits obtained from raw buckwheat flour, the analyzed Maillard reaction products were at a higher level than in the biscuits obtained from roasted flour. As was shown in our previous study [16,17] for the raw buckwheat the FAST index, soluble proteins, and browning index were 56.5, 31.9, and 0.27, respectively, and furosine was not detected in raw flour. While in roasted buckwheat flour the furosine, FAST index, soluble proteins, and browning index were significantly higher and amounted to 40.7, 294.2, 30.5, and 0.34, respectively.

The research showed the presence of furosine in biscuits obtained from fermented buckwheat flour as well as in samples from unfermented flour. This compound is a marker of the early-stage Maillard reaction and its presence in the analyzed biscuits is undesirable. Compared to control biscuits, both raw and roasted, a significantly higher content of furosine was noticed in biscuits obtained from fermented flour. Generally, after in vitro digestion, in both control and fermented samples, a significant increase in furosine content was found compared to undigested ones. Fermentation of raw flour by fungi did not influence the potential bioavailability of furosine, but for roasted flour, a significant decrease was noticed (Table 1). Tekliye et al. [18] presented that furosine may be a good indicator of the degree of Maillard reaction-induced damage during the processing of fermented milk. Yiltirak et al. [19] showed that compared to other grains (wheat, barley, rye, einkorn wheat, oat) buckwheat Maillard reaction products were considerably low, and this difference was also apparent after sprouting and it was probably attributed to the presence of high amounts of rutin. Zieliński et al. [20] found a decrease in the furosine content for muffins with the fermented buckwheat flour suspension compared to the muffins with the unfermented buckwheat flour suspension. The soluble protein content in biscuits obtained from fermented flours (both) was higher than in control biscuits. The in vitro digestion process increases the amount of soluble protein compared to the undigested sample (Table 1), and a high PB was noticed for analyzed samples.

Based on the analysis of the products of the advanced stage of the Maillard reaction, it was possible to calculate the nutritional value of the buckwheat biscuits in the form of the so-called FAST index (Fluorescent of Advanced Maillard products and Soluble Tryptophan). The calculated FAST index values for biscuits obtained from fermented raw and roasted buckwheat flour were 504 and 445%, respectively. These values were almost 2-times higher compared to control samples. Using the interpretation of the FAST index, i.e., the lower the value of the FAST index, the higher the nutritional value of the products, it was possible to find a diversified effect of fermented buckwheat flour on the nutritional value of biscuits. In this context, the nutritional value, both before and after in vitro digestion, was rather low for biscuits baked from buckwheat flour fermented with *Rhizopus oligosporus* 2740 (Table 1). It should also be noted that the use of fermented buckwheat flour to bake the biscuits did not increase the nutritional value in comparison to the control sample in the context of the FAST index analysis. The level of potential bioavailability obtained for this parameter was the lowest compared to the other analyzed parameters.

The marker of the final stage of the Maillard reaction is the so-called browning index closely related to the level of high molecular weight melanoidin’s (Table 1). The beneficial presence of these polymeric compounds in food is related to their participation in shaping the sensory properties of food. The increase in the browning index values for the sample obtained from fermented flour, both raw and roasted was observed compared to the control sample. Digestion of biscuits obtained from fermented raw and roasted buckwheat flour led to an increase in the potential bioaccessibility of melanoidin (PB > 1), as the browning index of those samples was several times higher after digestion. It should be noted, however, that the browning index was also several times higher after digesting biscuits baked from both unfermented flours. Physico-chemical properties of the food system and the processing conditions used for food preparation strongly affect the relation between color changes due to non-enzymatic brownings, such as the Maillard reaction, and the formation of compounds with antioxidant activity as presented by Manzocco et al. [21].

### 2.2. Phenolic Acids and Their Potential Bioaccessibility from Investigated Samples

**Figure 1:**
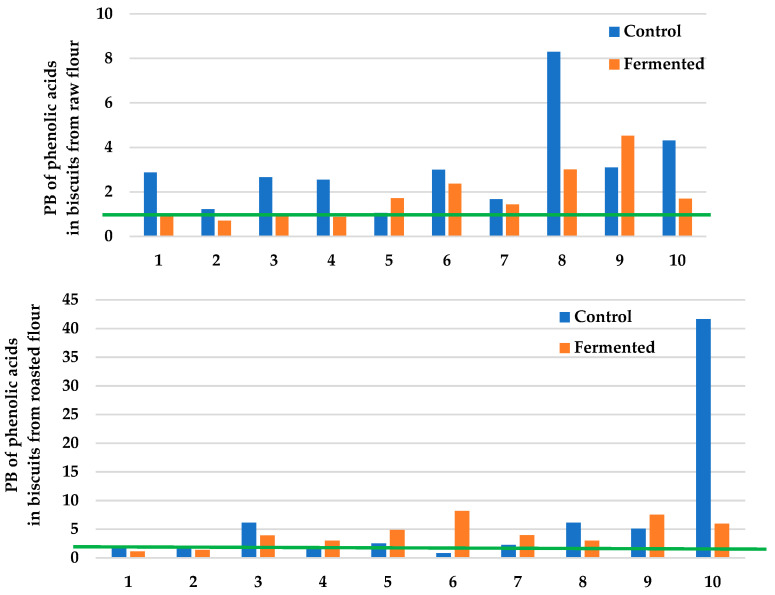
Potential bioaccessibility (PB) of phenolic acids from biscuits obtained from raw and roasted buckwheat flour. PB > 1: high bioaccessibility; PB < 1: low bioaccessibility. Phenolic acids: chlorogenic (1); p-coumaric (2); sinapic (3); ferulic (4); t-cinnamic (5); syringic (6); vanillic (7); isovanillic (8); protocatechuic (9); and caffeic (10).

The profile and the content of phenolic acids and their potential bioaccessibility were presented in Table 2 and Figure 1. The total phenolic content in buckwheat flour, raw and roasted, fermented with *Rhizopus oligosporus* 2710 was 1.34 and 2.30 mg GAE/g d.m., respectively [22]. In buckwheat flours and biscuits before and after in vitro digestion phenolic acids, such as ferulic, syringic, vanillic, protocatechuic, p-coumaric, caffeic, t-cinnamic, sinapic, chlorogenic, and isovanillic were detected. Among them, ferulic, syringic, vanillic, and coffee acids were dominant. Generally, fermentation by *Rhizopus oligosporus* 2710 has a different effect on the content of phenolic acids in buckwheat flour, both raw and roasted. The baking process used (220 °C/30 min) decreased the content of the analyzed phenolic acids, for biscuits obtained from raw and roasted flours. After in vitro digestion, a significant increase in the content of analyzed phenolic acid was found compared to undigested biscuits (Table 2). Generally, the potential bioaccessibility of phenolic acids from biscuits obtained from fermented flour was lower compared to control samples. The profile of phenolic acids in the investigated samples follows the data provided by other authors [23,24]. Furthermore, a decrease in the content of individual phenolic acids as a result of the roasting or baking process was in agreement with the data presented in the literature [24]. Noteworthy is the fact that phenolic acids are released during the digestion process, as evidenced also by the values of potential bioaccessibility, but lower for flour fermented by *Rhizopus oligosporus* 2710. Zhang et al. [25] showed also a positive correlation between the antioxidant activity and prebiotic effect of phenolic compounds in oat bran, which can be used to regulate the gut microbiota composition. Limited data on the bioaccessibility and bioavailability of cereal polyphenols and their interaction with the intestinal barrier, gut microbiome, and plasma inflammatory mediators were shown in the review by Ed Nignpense et al. [26]. The authors note that polyphenol bioaccessibility is low but dependent on the type of polyphenol or cereal matrix involved.

### 2.3. Flavonoids and Their Potential Bioaccessibility from Investigated Samples

**Figure 2:**
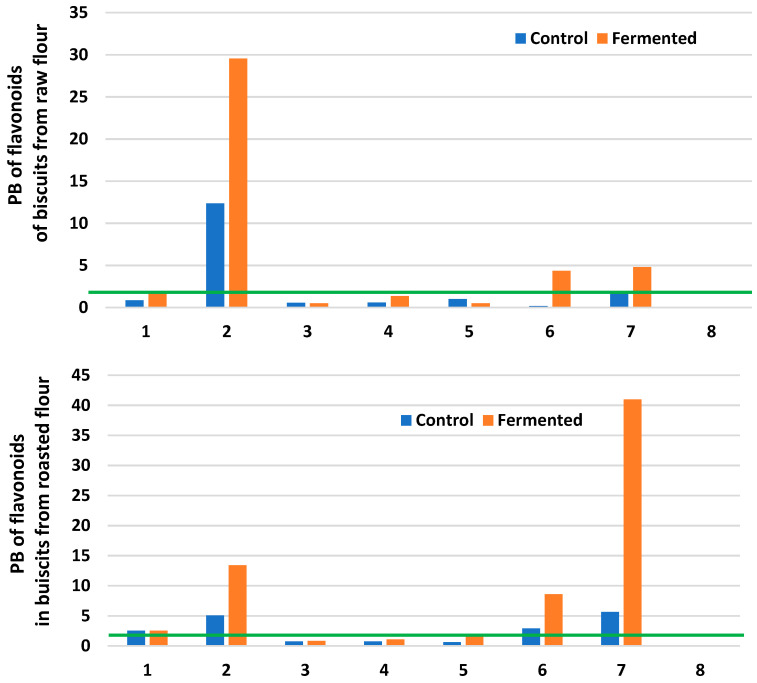
Potential bioaccessibility (PB) of flavonoids from biscuits obtained from raw and roasted buckwheat flour. PB > 1: high bioaccessibility; PB < 1: low bioaccessibility. Flavonoids: luteolin (1); kaempferol (2); vitexin (3); apigenin (4); orientin (5); epicatechin (6); quercitin (7); rutin (8).

The profile and the content of flavonoids and their potential bioaccessibility from the investigated samples were presented in Table 3 and Figure 2. The total flavonoid content in buckwheat flour, raw and roasted, fermented with *Rhizopus oligosporus* 2710 was 0.60 and 0.33 mg GAE/g d.m., respectively [22]. The dominant flavonoids in buckwheat products were rutin, epicatechin, vitexin, orientin, and quercetin, but also kaempferol, apigenin, and luteolin were present. The content of all flavonoids was higher in raw and fermented flours compared to roasted ones. Generally, it was found that the fermentation process decreases the content of the flavonoids, for both raw and roasted flour. Similar conclusions can be made regarding the baking process used. However, the digestion process of the biscuits caused the release of flavonoids and an increase in their content compared to non-digestible cookies was observed (Table 3). Generally, the potential bioaccessibility of flavonoids from buckwheat biscuits obtained from flour fermented by *Rhizopus oligosporus* 2710 was higher than from the control samples, both raw and roasted. The decrease in rutin content in buckwheat groat fermented by *Rhizopus oligosporus* compared to non-fermented samples was reported in our earlier work [7]. While Starzyńska-Janiszewska et al. [27] found a significant increase in rutin content in quinoa fermented with *R. oligosporus* compared to cooked quinoa seeds. The content of rutin and quercitin, and their bioaccessibility in raw and roasted buckwheat flour and biscuits obtained from flour fermented by different *Lactobacillus* strains were described by Zieliński et al. [10]. The bioaccessibility of rutin from biscuits was low contrary to results obtained for quercitin for which the index was greater than 1. Choi et al. [28] presented the improvement of flavonoids’ bioaccessibility upon baking and digestion which implies that buckwheat flavonoids are easily released from the food matrix.

## 3. Experimental

### 3.1. Chemicals

Reagents in MS grade, including acetonitrile, methanol, water, and formic acid were purchased from Sigma Chemical Co. (St. Louis, MO, USA). While diethyl ether (Et2O), hydrochloric acid (HCl), and sodium hydroxide (NaOH) were obtained from POCH S.A. (Gliwice, Poland). Compounds standard (phenolic acids, flavonoids) were from Sigma Chemical Co. (St. Louis, MO, USA) and were used for identification and calculation.

### 3.2. Buckwheat Flour

Commercial Polish common buckwheat flour (*Fagopyrum esculentum* Moench) and roasted buckwheat groats were purchased from local industry (Melvit S.A., Kruki, Poland). Roasted buckwheat groats were ground in a laboratory mill equipped with screens of different diameters of holes. According to the produced declaration, the starch, proteins, and ash content of buckwheat flour were 58.5 ± 0.3%; 19.2 ± 0.1%, and 3.2 ± 0.4% on a dry basis, respectively. The starch, proteins, and ash content of roasted buckwheat flour were 58.5 ± 0.3%; 19.2 ± 0.1%, and 3.2 ± 0.4% on a dry basis, respectively.

### 3.3. Pretreatment of Buckwheat Flour

Before the fermentation process, buckwheat flour (raw or roasted) was pretreated. About 50 g of each type of flour was suspended with 950 mL of distilled water. Next, the suspension was well stirred during heating at 90 °C for 45 min., then autoclaved at 121 °C/15 min. and finally cooled to 37 °C. The pretreatment was carried out to reduce microbial populations existing on buckwheat flours before inoculated fermentation since they would compete with and inhibit the growth of inoculated microbes during the fermentation process.

### 3.4. Fermentation of Buckwheat Flours by Rhizopus oligosporus 2710, Preparation of Buckwheat Biscuits from Fermented Flours, and In Vitro Digestion

#### 3.4.1. Fermentation of Buckwheat Flour

The 5% suspension of pretreated buckwheat flour in distilled water was inoculated with *Rhizopus oligosporus* 2710 filamentous fungus at the level of 10^5^ CFU/mL. Fermentation of buckwheat flour suspension was carried out at 37 °C for 24 h. The fermentation was described in detail by Wronkowska et al. [17]. The *Rhizopus oligosporus* 2710 filamentous fungus originated from ATCC^®^. After fermentation, the samples were freeze-dried (Christ—Epsilon 2-6D LSC plus, Osterode am Harz, Germany).

#### 3.4.2. Preparation of Buckwheat Biscuits from Fermented Flour

The biscuit dough was prepared according to the AACC 10–52 method [29], with the modification proposed by Hidalgo and Brandolini [30]. The dough was cut with a square cookie cutter (60 mm). Biscuits were baked at 220 °C for 30 min (electric oven DC-21 model, Sveba Dahlen AB, Fristad, Sweden). The control biscuits were formulated on non-fermented buckwheat flour (raw or roasted). The buckwheat biscuits were lyophilized, milled, and stored in a refrigerator until analysis.

#### 3.4.3. In Vitro Digestion of Buckwheat Biscuits

Buckwheat biscuits were in vitro digested as described by Delgado-Andrade et al. [31] with some modifications [22]. Briefly, lyophilized and milled buckwheat biscuits were suspended in deionized water then an α-amylase solution was added to the samples. Samples were shaken in a water bath at 37 °C for 30 min. For the gastric digestion, the pH was reduced to 2.0, pepsin solution was added and the incubation was continued under the same conditions for 120 min. In the next step, the pH was adjusted to 6.0, and a mixture of pancreatin and bile salts extract was added. Subsequently, the pH was increased to 7.5 and the samples were incubated at 37 °C for 120 min. After incubation, the digestive enzymes were inactivated by heating at 100 °C for 4 min and cooled for centrifugation. The supernatants obtained were stored at −18 °C.

### 3.5. Maillard Reaction Products Determination

Material for the analysis of furosine, FAST index, and browning was prepared as follows: dry samples were mixed with 6% of aqueous sodium dodecyl sulfate, incubated for 30 min with stirring every 10 min for 30 s, and filtered, and then the filtrates were used for the analysis. All assays were made according to procedures described in detail by Wronkowska et al. [32]. The content of soluble proteins was determined according to Wronkowska et al. [17]. Evaluation of the potential bioaccessibility (PB) in vitro of all analyzed Maillard reaction products from biscuits was performed according to Zieliński et al. [9]. It was calculated by dividing the amount of the analyzed parameter after digestion by the amount of this parameter before digestion. The obtained values above one indicate high bioaccessibility, and values below one—low bioaccessibility.

### 3.6. Extraction and Isolation of the Main Phenolic Compounds and Flavonoids from Analyzed Samples

The profile and content of phenolic acids and flavonoids were analyzed according to the method described by Jeż et al. [33] and Płatosz et al. [34], and for the determination, the system HPLC-MS/MS involving a HALO column was applied. Briefly, samples were extracted with a mixture of methanol/water/formic acid by stirring overnight. Then, after centrifugation, the obtained supernatants were stored at −80 °C until HPLC analysis. The profile and content of phenolic acids and flavonoid forms (free, esters, and glycosides) were analyzed. Therefore, free forms of phenolic acids and flavonoids were isolated with diethyl ether after acidification to pH 2. Esters of phenolic compounds were hydrolyzed with 4 M NaOH, whereas glycosides were hydrolyzed with 6 M HCl. Next, the extracts were evaporated to dryness under a nitrogen atmosphere. Finally, the dry residues obtained from analyzed samples were dissolved in methanol, centrifuged, and analyzed by using the HPLC system (LC-200, Eksigent, Vaughan, ON, Canada) coupled with a mass spectrometer (QTRAP 5500, AB Sciex, Vaughan, ON, Canada) consisting of a triple quadrupole, ion trap, and ion source of electrospray ionization (ESI). The chromatographic separation was conducted with a HALO C18 column (Eksigent, Vaughan, ON, Canada). The elution was conducted using a solvent gradient system consisting of solvent A (0.9% (*v*/*v*) formic acid aqueous solution) and solvent B (0.9% (*v*/*v*) formic acid acetonitrile solution). Identification and quantitation of the phenolic acids and flavonoids were based on the comparison of their retention times and the presence of the respective parent and daughter ion pairs (Multiple Reaction Monitoring method, MRM) with data obtained after analysis of the authentic standards. The external standards (0.01–0.5 g/mL) had linear calibration curves with a coefficient of determination of 0.997–0.999. The content of individual phenolics was expressed as the content of their free and conjugated forms (a sum of phenolics released from ester and glycosidic bonds) of phenolic acids or flavonoids. The results were expressed in g/g dry matter (dm) of analyzed samples.

The potential bioaccessibility (PB) was calculated by dividing the amount of the analyzed parameter after digestion by the amount of this parameter before digestion. A PB value higher than one indicates high bioaccessibility and a PB value lower than one indicates low bioaccessibility.

## 4. Conclusions

Biscuits formulated from common buckwheat flours, raw and roasted, and fermented by *Rhizopus oligosporus* 2710 were used in this study. The potential bioaccessibility (PB) of phenolic compounds, flavonoids, and selected Maillard reaction products (furosine, FAST index, and the level of melanoidins defined by the browning index) after in vitro digestion was determined. Generally, compared to the control biscuits prepared from non-fermented flours higher levels of all analyzed Maillard products were found in the biscuits obtained from fermented flours. After in vitro digestion, the highest amount of all analyzed Maillard products was found for all samples obtained from fermented flours, which resulted in high values of their potential bioaccessibility. The presence of the ten phenolic acids (ferulic, syringic, caffeic, sinapic, *para*-coumaric, chlorogenic, trans-cinnamic, protocatechuic, vanillic, and isovanillic) and the eight flavonoids (rutin, epicatechin, vitexin, orientin, quercetin, kaempferol, apigenin, and luteolin) in biscuits obtained from non-fermented and fermented buckwheat flours was noticed. Generally, after in vitro digestion the highest content of phenolic acid was found in all analyzed biscuits, which was connected with high values of their potential bioaccessibility. However, in the case of flavonoids, the influence of the fermentation process is visible, because the PB values were generally higher for biscuits obtained from both fermented flours. Fermentation of buckwheat flours with *Rhizopus oligosporus* seems to be a good way to obtain high-quality biscuits however further research on their functional properties is needed.

## Figures and Tables

**Table 1:** The Maillard reaction products and their potential bioaccessibility in biscuits from raw and roasted buckwheat before and after in vitro digestion.

Sample	Maillard Reaction Products	Biscuits	Digested Biscuits	PB
Raw				
Control	Furosine (mg/g DM)	3.28 ± 0.04 bB	7.53 ± 0.51 bA	2.3
Fermented		20.27 ± 0.07 aB	41.09 ± 2.74 aA	2.0
Control	FAST index (%)	262.2 ± 18.4 bA	55.7 ± 2.1 bB	0.2
Fermented		504.8 ± 40.4 aA	203.0 ± 23.5 aB	0.4
Control	Soluble proteins (mg/g DM)	41.05 ± 0.23 bB *	99.03 ± 5.47 bA	2.4
Fermented		115.14 ± 0.42 aB	225.29 ± 16.46 aA	1.9
Control	Browning index (AU)	0.21 ± 0.01 bB	0.96 ± 0.02 bA	4.6
Fermented		1.05 ± 0.19 aB	3.34 ± 0.23 aA	3.2
Roasted				
Control	Furosine (mg/g DM)	1.06 ± 0.04 bB	5.20 ± 0.83 bA	4.9
Fermented		18.41 ± 0.06 aB	35.31 ± 2.55 aA	1.9
Control	FAST index (%)	214.0 ± 4.6 bA	55.0 ± 0.5 bB	0.3
Fermented		445.3 ± 9.3 aA	117.0 ± 6.4 aB	0.3
Control	Soluble proteins (mg/g DM)	29.26 ± 0.17 bB	68.53 ± 3.80 bA	2.3
Fermented		62.68 ± 0.89 aB	170.09 ± 3.23 aA	2.7
Control	Browning index (AU)	0.32 ± 0.01 bB	1.09 ± 0.04 aA	3.4
Fermented		0.50 ± 0.05 aB	1.97 ± 0.07 aA	3.9

Data expressed as mean ± standard deviation (*n* = 3). DM: dry matter. Values followed by the same letter in the same column (a,b) or raw (A,B) are not significantly different at a 95% confidence level. PB—potential bioaccessibility. Browning is expressed in arbitrary units (AU). * Wronkowska et al. [17].

**Table 2:** The profile and content of phenolic acids (μg/g DM) and their potential bioaccessibility in raw and roasted buckwheat: flour, biscuits before and after in vitro digestion.

Sample	Phenolic Acids	Flour	Biscuits	Digested Biscuits	PB
Raw					
Control	Ferulic	122.67 ± 4.12 aA	3.21 ± 0.13 bC	8.17 ± 0.21 aB	2.5
Fermented		78.05 ± 2.08 bA	5.98 ± 0.16 aB	5.22 ± 0.14 bB	0.9
Control	Syringic	78.80 ± 0.85 aB	43.63 ± 1.33 aC	130.65 ± 1.22 aA	3.0
Fermented		41.82 ± 1.60 bB	37.16 ± 1.12 bB	88.06 ± 1.80 bA	2.4
Control	Vanillic	55.52 ± 2.08 bC	112.66 ± 2.66 aB	187.90 ± 18.83 aA	1.7
Fermented		94.83 ± 2.55 aAB	80.12 ± 2.68 bB	114.94 ± 4.87 bA	1.4
Control	Protocatechuic	26.57 ± 0.78 aC	65.79 ± 2.46 aB	203.57 ± 6.15 aA	3.1
Fermented		20.44 ± 0.68 aB	27.40 ± 1.28 bB	124.06 ± 0.71 bA	4.5
Control	p-Coumaric	22.13 ± 0.24 aA	21.53 ± 3.10 bA	26.33 ± 0.14 aA	1.2
Fermented		13.72 ± 0.57 bC	32.66 ± 0.44 aA	22.80 ± 0.42 aB	0.7
Control	Caffeic	21.80 ± 0.08 bA	3.40 ± 0.11 bC	14.64 ± 0.09 bB	4.3
Fermented		46.88 ± 0.98 aA	12.47 ± 0.38 aC	21.15 ± 0.36 aB	1.7
Control	t-Cinnamic	7.08 ± 0.10 aB	7.86 ± 0.02 aB	8.29 ± 0.02 aA	1.1
Fermented		0.33 ± 0.02 bC	2.82 ± 0.08 bB	4.84 ± 0.16 bA	1.7
Control	Sinapic	6.00 ± 0.33 bB	8.33 ± 0.10 bB	22.16 ± 0.30 aA	2.7
Fermented		9.14 ± 0.27 aB	17.84 ± 1.42 aA	16.90 ± 1.54 bA	0.9
Control	Chlorogenic	0.04 ± 0.00 bA	0.08 ± 0.00 aA	0.23 ± 0.10 aA	2.9
Fermented		0.27 ± 0.00 aA	0.09 ± 0.00 aA	0.09 ± 0.00 bA	1.0
Control	Isovanillic	n.d.	2.10 ± 0.05 bB	17.41 ± 0.37 aA	8.3
Fermented		n.d.	5.96 ± 0.34 aB	17.93 ± 0.09 aA	3.0
Roasted					
Control	Ferulic	23.50 ± 2.43 bA	2.55 ± 0.07 aC	4.93 ± 0.12 bB	1.9
Fermented		30.55 ± 3.00 aA	2.52 ± 0.06 aC	7.49 ± 0.19 aB	3.0
Control	Syringic	24.66 ± 0.74 bC	100.93 ± 2.05 aA	80.86 ± 4.29 bB	0.8
Fermented		86.90 ± 1.60 aB	11.55 ± 0.31 bC	94.21 ± 2.49 aA	8.2
Control	Vanillic	68.62 ± 1.07 bB	49.74 ± 2.19 aC	111.25 ± 4.11 bA	2.2
Fermented		187.76 ± 6.25 aA	31.41 ± 0.90 bC	123.68 ± 2.70 aB	3.9
Control	Protocatechuic	25.24 ± 0.32 bB	29.96 ± 0.57 aB	151.53 ± 4.45 bA	5.1
Fermented		31.28 ± 0.57 aB	25.03 ± 0.70 bC	188.21 ± 2.67 aA	7.5
Control	p-Coumaric	13.52 ± 0.13 bA	7.53 ± 0.34 bB	14.94 ± 0.17 bA	2.0
Fermented		31.00 ± 2.13 aA	13.28 ± 0.10 aC	18.46 ± 0.22 aB	1.4
Control	Caffeic	89.33 ± 3.82 aA	0.70 ± 0.00 bC	29.13 ± 0.79 bB	41.6
Fermented		70.15 ± 0.56 bA	6.04 ± 0.17 aC	35.85 ± 0.51 aB	5.9
Control	t-Cinnamic	9.98 ± 0.19 aA	2.52 ± 0.05 aC	6.32 ± 0.06 bB	2.5
Fermented		0.54 ± 0.03 bC	1.60 ± 0.06 bB	7.80 ± 0.06 aA	4.9
Control	Sinapic	8.19 ± 0.55 aB	1.97 ± 0.03 bC	12.07 ± 0.30 bA	6.1
Fermented		6.14 ± 0.07 bB	3.60 ± 0.12 aC	13.99 ± 0.40 aA	3.9
Control	Chlorogenic	0.07 ± 0.00 aA	0.06 ± 0.00 aA	0.11 ± 0.00 aA	1.8
Fermented		0.17 ± 0.00 aA	0.10 ± 0.00 aA	0.11 ± 0.00 aA	1.1
Control	Isovanillic	n.d.	3.12 ± 0.17 bB	19.09 ± 3.74 aA	6.1
Fermented		n.d.	4.87 ± 0.23 aB	14.48 ± 0.29 bA	3.0

Data expressed as mean ± standard deviation (*n* = 3). DM: dry matter. PB—potential bioaccessibility. Values followed by the same letter in the same column (a,b) or raw (A–C) are not significantly different at a 95% confidence level.

**Table 3:** The profile and total content of flavonoids (μg/g DM) and their potential bioaccessibility in raw and roasted buckwheat: flour, biscuits before and after in vitro digestion.

Sample	Flavonoids	Flour	Biscuits	Digested Biscuits	PB
Raw					
Control	Rutin	376.40 ± 6.30 aA *	90.53 ± 3.45 bB *	1.90 ± 0.07 bC *	0.02
Fermented		367.80 ± 1.80 aA	150.77 ± 5.09 aB	2.23 ± 0.04 aC	0.01
Control	Epicatechin	183.33 ± 0.64 aA	91.69 ± 2.73 aB	16.45 ± 0.53 aC	0.2
Fermented		64.34 ± 0.08 bA	2.39 ± 0.07 bC	10.40 ± 0.24 bB	4.4
Control	Vitexin	21.24 ± 1.00 aA	15.04 ± 0.21 aB	8.30 ± 0.29 aC	0.6
Fermented		10.29 ± 0.58 bB	13.51 ± 0.35 bA	6.58 ± 0.19 bC	0.5
Control	Orientin	17.62 ± 0.25 bA	4.21 ± 0.18 aB	4.23 ± 0.04 aB	1.0
Fermented		20.08 ± 0.16 aA	4.47 ± 0.18 aBC	2.23 ± 0.05 bC	0.5
Control	Quercitin	8.32 ± 0.02 aA *	4.55 ± 0.25 aC *	7.55 ± 0.28 aB *	1.7
Fermented		3.80 ± 0.01 bB	1.41 ± 0.06 bC	6.77 ± 0.20 bA	4.8
Control	Kaempferol	1.12 ± 0.02 aB	0.75 ± 0.12 aC	9.27 ± 0.08 bA	12.4
Fermented		0.31 ± 0.07 bB	0.35 ± 0.09 bB	10.34 ± 0.26 aA	29.5
Control	Apigenin	0.30 ± 0.04 bC	2.13 ± 0.20 aA	1.25 ± 0.02 aB	0.6
Fermented		2.35 ± 0.02 aA	0.92 ± 0.03 bC	1.24 ± 0.01 aB	1.3
Control	Luteolin	0.26 ± 0.02 aA	0.22 ± 0.02 aA	0.19 ± 0.02 aA	0.9
Fermented		0.09 ± 0.01 bB	0.11 ± 0.04 bA	0.18 ± 0.08 aA	1.6
Roasted					
Control	Rutin	220.40 ± 0.30 *	61.00 ± 3.04 *	2.51 ± 0.10 *	0.04
Fermented		150.60 ± 6.90	92.60 ± 6.71	5.19 ± 0.04	0.06
Control	Epicatechin	138.48 ± 0.77 aA	6.25 ± 0.20 aC	18.22 ± 0.51 aB	2.9
Fermented		86.85 ± 0.36 bA	2.18 ± 0.10 bC	18.74 ± 0.43 aB	8.6
Control	Vitexin	22.41 ± 1.22 aA	10.26 ± 0.04 aB	7.56 ± 0.15 aC	0.7
Fermented		11.31 ± 0.61 bA	9.23 ± 0.17 aB	7.75 ± 0.20 aC	0.8
Control	Orientin	14.19 ± 0.45 aA	5.29 ± 0.01 aB	3.31 ± 0.06 aB	0.6
Fermented		8.04 ± 0.40 bA	1.54 ± 0.05 bC	2.88 ± 0.08 aB	1.9
Control	Quercitin	3.82 ± 0.04 aB *	1.37 ± 0.01 aC *	7.72 ± 0.45 bA *	5.6
Fermented		2.80 ± 0.01 bB	0.29 ± 0.01 bC	11.88 ± 0.19 aA	41.0
Control	Kaempferol	0.78 ± 0.01 aC	3.39 ± 0.27 aB	17.26 ± 0.54 bA	5.1
Fermented		0.40 ± 0.02 bC	1.41 ± 0.21 bB	18.87 ± 0.42 aA	13.4
Control	Apigenin	0.71 ± 0.00 bC	1.54 ± 0.05 aA	1.16 ± 0.02 aB	0.8
Fermented		0.95 ± 0.02 aB	1.08 ± 0.02 bB	1.16 ± 0.02 aA	1.1
Control	Luteolin	0.24 ± 0.01 aB	0.15 ± 0.03 aC	0.38 ± 0.03 aA	2.5
Fermented		0.08 ± 0.01 bC	0.15 ± 0.01 aB	0.38 ± 0.01 aA	2.5

Data expressed as mean ± standard deviation (*n* = 3). DM: dry matter. PB—potential bioaccessibility. Values followed by the same letter in the same column (a,b) or raw (A–C) are not significantly different at a 95% confidence level. * data were presented by Zieliński et al. [10].

## Data Availability

Data sharing is not applicable.

## Sample Availability

Samples of the compounds are not available from the authors.
